# Gut Microbiota in Untreated Diffuse Large B Cell Lymphoma Patients

**DOI:** 10.3389/fmicb.2021.646361

**Published:** 2021-04-13

**Authors:** Li Yuan, Wei Wang, Wei Zhang, Yan Zhang, Chong Wei, Jingnan Li, Daobin Zhou

**Affiliations:** ^1^Department of Hematology, Peking Union Medical College Hospital, Peking Union Medical College, Chinese Academy of Medical Sciences, Beijing, China; ^2^Department of Gastroenterology, Peking Union Medical College Hospital, Peking Union Medical College, Chinese Academy of Medical Sciences, Beijing, China

**Keywords:** hematologic malignancies, diffuse large B cell lymphoma, gut microbiota, proteobacteria, 16S rRNA

## Abstract

Intestinal microecology plays an important role in the development and progression of hematological malignancies. However, characteristics of gut microbiota in diffuse large B cell lymphoma (DLBCL) have not been reported. The microbiota composition of fecal samples from 25 untreated DLBCL patients and 26 healthy volunteers was examined by 16S rRNA gene sequencing. On α-diversity analysis, there was no significant difference in species diversity and abundance between the two groups. However, a significant difference was observed on β-diversity analysis. The intestinal microbiota in patients with DLBCL showed a continuous evolutionary relationship, which progressed from phylum, *proteobacteria*, to genus, *Escherichia-Shigella*. Their abundance was significantly higher than that of the control group. At the genus level, *Allisonella*, *lachnospira*, and *Roseburia* were more abundant in patients with DLBCL than in the control group. Functional prediction by PICRUSt indicated that thiamine metabolism and phenylalanine, tyrosine, and tryptophan biosynthesis were significantly lower in the DLBCL group than in the control group. In conclusion, our results clearly demonstrate that the gut microbiota was changed significantly in DLBCL. The study highlights fundamental differences in the microbial diversity and composition of patients with DLBCL and paves the way for future prospective studies and microbiome-directed interventional trials to improve patient outcomes.

## Introduction

Intestinal microecology plays an important role in the occurrence and development of many hematological malignancies (Christopher et al., 2019; Lee et al., 2019; Pianko et al., 2019). Gut microbiota may induce gene mutations (Arabski et al., 2005), possibly through chronic inflammation to disrupt the balance between cell proliferation and apoptosis (Kenza et al., 2018; Kawari et al., 2019), and may initiate unnecessary innate and adaptive immune responses (Calcinotto et al., 2018). Gene mutations and aberrant immune responses contribute to the occurrence and development of hematological malignancies.

Diffuse large B cell lymphoma (DLBCL) is the most common type of adult non-Hodgkin’s lymphoma (NHL), and >60% of all adult NHLs were diagnosed as DLBCLs in China (Abid et al., 2005). Although the treatment remission rate has been increased, one-third of patients still do not benefit from the current treatment strategy (Roschewski et al., 2014). This is because the pathogenesis of DLBCL is not fully understood and the treatment is limited. With the rapid development of high-throughput analysis, we have gradually gained more insights into the pathogenesis of DLBCL. For example, whole genome sequencing has revealed that many genes, epigenetic molecules, and specific signaling pathways are altered in DLBCL, and these changes may promote the occurrence and development of DLBCL (Scott and Gascoyne, 2014; Younes et al., 2017). Many studies have also indicated that the gut microbiota may play an indispensable role in the pathogenesis of DLBCL.

The genome of the microbial community in the human gastrointestinal system is >150 times that of the human genome. These long-lived microorganisms and their metabolites easily interact with host cells (Qin et al., 2010). Therefore, the microbial genome in the human body is also called the second human genome. Various studies have demonstrated that the gut microbiota plays an important role in the occurrence and development of malignancies. However, there are few studies on the role of the gut microbiota in NHL, and the role of gut microbiota in DLBCL has not been reported.

The present study investigated the gut microbiota changes in patients with untreated DLBCL. More specifically, the gut microbiota in patients with DLBCL was examined by 16S rRNA and compared with that of healthy controls. Our results indicated that the gut microbiota has changed considerably in DLBCL patients. Exploring the role of microbiota alterations in the development of DLBCL may reveal novel biomarkers used for DLBCL diagnosis and prognostic prediction, which could lay a solid foundation for better management of DLBCL and drug development.

## Materials and Methods

### Study Subjects and Groups

Twenty-five patients with newly diagnosed untreated DLBCL (Lynch et al., 2017) and 26 matched healthy controls were recruited from November 2019 to August 2020 in the Department of Hematology of Beijing Union Hospital. The microbiota composition of fecal samples from these 51 cases was analyzed. All 25 patients were Chinese living in Mainland China, without chronic inflammatory diseases in the gastrointestinal tract and other tumors, and they did not receive treatment for DLBCL or antibiotic treatment within 4 weeks.

This study population was divided into the experimental group (EG) of untreated DLBCL patients and control group (CG) of healthy volunteers. The patients were further classified into the germinal center group (GCB DLBCL was similar to that of germinal center B cells) or activated B cell-like group (ABC DLBCL was induced by activation of peripheral blood B cells *in vitro*) with or without gastrointestinal involvement (GI or NGI).

### Sample Collection, DNA Extraction, Polymerase Chain Reaction, and Microbiota Analysis

The microbiota composition of fecal samples of 25 DLBCL patients and 26 healthy volunteers matched for the place of birth, age, and sex to that of DLBCL patients were analyzed. The inclusion criteria included: no history of tumor and chronic gastrointestinal inflammatory diseases, no use of antibiotics within 4 weeks, and no history of diarrhea within 2 weeks.

The above 51 participants gave signed informed consent for sample collection. When the samples were collected, each participant obtained a sterile plastic bag and a fecal collection tube with microbial culture medium, and the fresh feces were collected aseptically in the fecal collection tube. Immediately after sampling, the specimens were stored in a −20°C freezer. Within 24 h, they were sent to the Hematology Laboratory of Beijing Union Hospital for storage at −80°C until DNA was extracted.

Fecal DNA was extracted using PowerSoil DNA Isolation Kit (MoBio Laboratories, Carlsbad, CA, United States) following the recommended protocols. Purity and quality of the genomic DNA were checked on 1% agarose gels and a NanoDrop spectrophotometer (Thermo Scientific). The V3-4 hypervariable regions of bacterial 16S rRNA genes were amplified by polymerase chain reaction (PCR) with the primers 338F (ACTCCTACGGGAGGCAGCAG) and 806R (GGACTACHVGGGTWTCTAAT) (Munyaka et al., 2015). The PCR products were purified using an Agencourt AMPure XP Kit. Deep sequencing was performed on Miseq platform at Allwegene Company (Beijing, China). After the run, image analysis, base calling, and error estimation were performed using Illumina Analysis Pipeline version 2.6. The original sequence was uploaded to the SRA database of NCBI^1^. The raw data were first screened, and sequences were removed from further analysis if they were shorter than 230 bp, with a low-quality score (≤20) or contained ambiguous bases or did not exactly match to primer sequences and barcode tags. Qualified reads were separated using the sample-specific barcode sequences and trimmed with Illumina Analysis Pipeline version 2.6. The datasets were analyzed using QIIME. The sequences were clustered into operational taxonomic units (OTUs) at a similarity level of 97% (Edgar, 2013), to generate rarefaction curves and to calculate the richness and diversity indices. The Ribosomal Database Project (RDP) Classifier tool was used to classify all sequences into different taxonomic groups (Cole et al., 2009). QIIME1 version 1.8.0 software was used to analyze α-diversity index (including Shannon, Simpson, and Chao1 indexes), and comparison of α-diversity metrics was assessed using the Turkey test. Partial least squares discrimination analysis (PLS-DA) was used to analyze β-diversity using R version 3.6.0. The differences between groups of metastats were analyzed by software mothur version 1.34.4 (Jami et al., 2013). Linear discriminant analysis effect size was analyzed by phython version 2.7 to show the diversity between EG and CG. Comparison of β-diversity metrics was assessed using the Wilcoxon rank sum test, Kruskal-Wallis test, and analysis of similarities. The correlation between the diversity of gut microbiota and physiological indexes (age, IPI score, and LDH) was evaluated by receiver operating characteristic curve using R language (PROC package). The PICRUSt software was used to predict the function of gut microbiota from 16S sequence results, and the functional changes among groups were analyzed based on the prediction results (Langille et al., 2013).

## Results

### Baseline Characteristics of the Study Population

The study included 25 untreated DLBCL patients (EG), with 12 (48%) men and 13 (52%) women. The median age was 58 years (range, 22–76 years). These 25 DLBCL patients were grouped into GCB (14 cases, 58.3%) or ABC (10 cases, 41.7%) types according to pathological subtypes. Two (8%) of them received proton pump inhibitors (PPIs) for digestive symptoms within 4 weeks, and the others did not (23 cases, 92%). One patient could not be classified based on pathology. Eight (32%) patients were GI and 17 (68%) were NGI. At the time of diagnosis, the prognosis of these 25 patients was assessed according to the IPI score (0–5) (Table 1). The median age of the CG was 56 years old (range, 24–78 years) with 14 (53.8%) men and 12 (46.2%) women. There was no significant difference in age and gender between the EG and the CG (*P* = 0.93 and 0.68, respectively). Baseline characteristics of the cohort are listed in Table 1.

**TABLE 1:** Baseline characteristics of patients and controls.

	Patient group (*n* = 25)	Control group (*n* = 26)
Median age, yr	58	56
**Gender, *n* (%)**		
Male	12 (48)	14 (53.8)
Female	13 (52)	12 (46.2)
Race/Region, *n*		
East Asian/China	25	26
**Pathological subtype, *n* (%)**		
GCB	14 (56)	
ABC	10 (40)	
Unclassifiable	1 (4)	
**Origin involved, *n* (%)**		
GI	8 (32)	
NGI	17 (68)	
**IPI score, *n* (%)**		
0	6 (24)	
1	5 (20)	
2	5 (20)	
3	5 (20)	
4	2 (8)	
5	2 (8)	
Median lactate dehydrogenase (range) IU/L	324.32 (116–1,510)	
**PPIs**		
Received	2 (8)	
Not received	23 (92)	

### Microbiota Distributions in the Untreated DLBCL Patients and Healthy Controls

#### OTU Distribution and OTU Notes of Untreated DLBCL Patients and Healthy Controls

**FIGURE 1:**
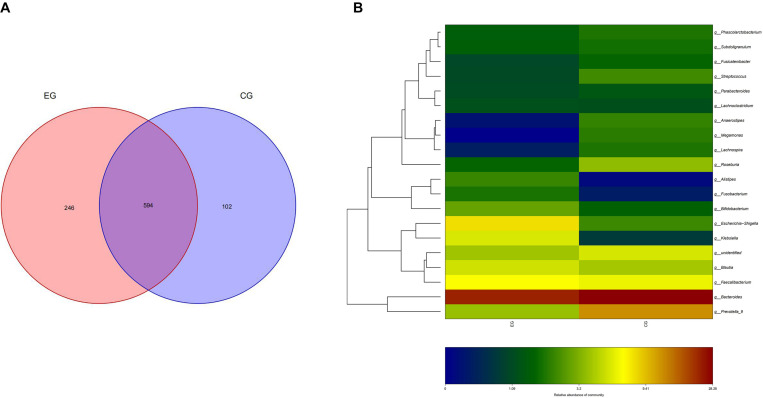
OTU distribution and OTU notes of EG and CG. **(A)** Number of OTUs shared by both groups and unique to each group is represented by Venn diagram. **(B)** Cluster analysis of species/OTU relationship at genus level. The horizontal ordinate is the name of the two groups, and the longitudinal ordinate is the name of species/OTU at genus level. The depth of color represents the level of OTU/species.

A total of 51 fecal specimens from the EG and CG were analyzed by 16S rRNA gene sequencing. The optimized sequences were obtained through data quality control, sequential splicing, filtering, and removing the chimera. The length of the optimized sequences was mostly enriched at 400–440 bp, and 4,666,988 reads were obtained and 39,712 reads were achieved per one sample. In 51 sequenced stool samples, 840 OTUs were identified and classified into 12 phyla, 22 classes, 31 orders, 56 families, 201 genera, and 43 species. There were 840 and 696 OTUs in the EG and CG, respectively, with 594 identical in both groups (Figure 1).

#### α-Diversity and β-Diversity Analysis in EG and CG

**FIGURE 2:**
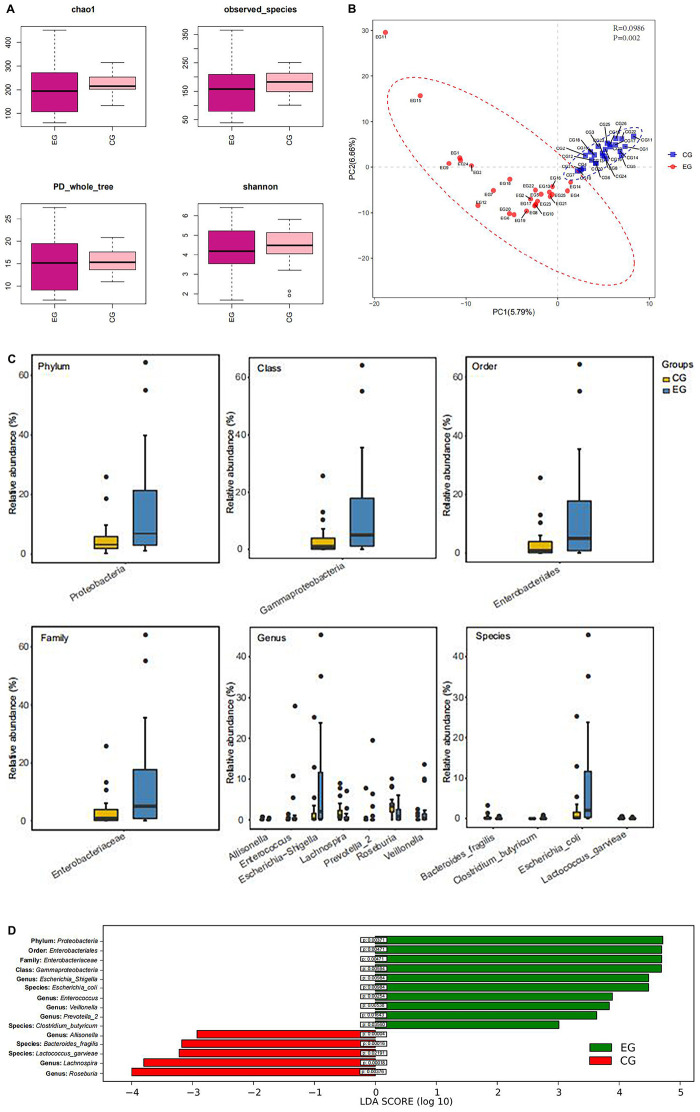
Box diagram of α-diversity index and β-diversity analysis. **(A)** Evenness and species richness of fecal samples in EG and CG. **(B)** OTU-based partial minimum multiplicative discriminant analysis showed that there were significant differences between the groups. **(C)** Difference of flora in phylum, class, order, family, genus, and species between the groups. **(D)** LDA displays species with significant differences in abundance between two groups.

Shannon-Wiener curves were used to assess the adequacy of stool samples in the EG and CG. The curve is flat, which indicated that the amount of sequencing data is sufficient to reflect most microbial information in the sample. α-Diversity analysis indicates that there was no significant difference in species diversity and abundance between EG and CG (chao1 index *P* = 0.43, Shannon index *P* = 0.69, observed_species index *P* = 0.35, PD_whole_tree index *P* = 0.70) (Figure 2A). However, a significant difference was found between the EG and the CG (*P* = 0.002) by β-diversity analysis (Figure 2B). The abundance of the *Proteobacteria* (phylum) (*P* = 0.0037), *Gammaproteobacteria* (class) (*P* = 0.0059), *Enterobacteriales* (order) (*P* = 0.0047), and *Enterobacteriaceae* (family) (*P* = 0.0047) of the EG was significantly higher than that of the CG. At the genus level, the abundance of *Escherichia-Shigella* (*P* = 0.009), *Enterococcus* (*P* = 0.0027), *Veillonella* (*P* = 0.0049), and *Prevotella-2* (*P* = 0.016) was significantly higher in EG. At the species level, *Escherichia coli* (*P* = 0.011) and *Clostridium butyricum* (*P* = 0.035) increased significantly in the EG. However, at the genus level, the abundance of *Allisonella* (*P* = 0.00004), *Lachnospira* (*P* = 0.00018), and *Roseburia* (*P* = 0.003) was significantly higher in the CG. At the species level, *Bacteroides fragilis* (*P* = 0.0002), *Lactococcus garvieae* (*P* = 0.03) predominated in the CG (Figures 2C,D).

**FIGURE 3:**
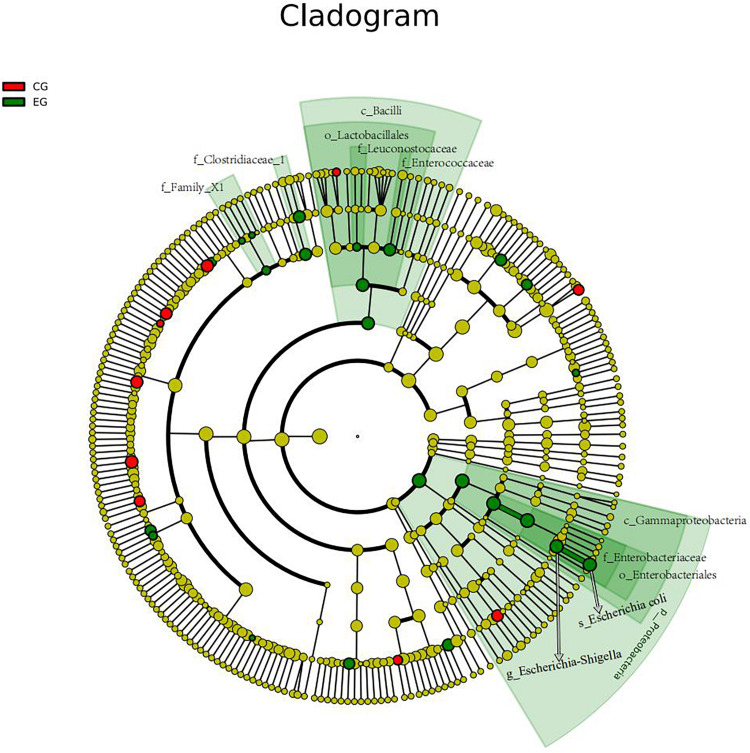
The gut microbiota of the untreated DLBCL patients showed a continuous evolutionary relationship at six levels: *Proteobacteria* (phylum), *Gammaproteobacteria* (class), *Enterobacteriales* (order), *Enterobacteriaceae* (family), *Escherichia-Shigella* (genus), and *E. coli* (species). The circles radiating from the inside to the outside represent the taxonomic level from phylum to species. Each small circle in different classification level represents a classification at this level, and the diameter of small circle is directly proportional to the relative abundance. The species with no significant difference were uniformly colored yellow. The red nodes indicated the microbial groups that played an important role in the CG, and the green nodes showed the microbial groups that played an important role in the EG.

The intestinal microbiota in patients with untreated DLBCL showed a continuous evolutionary relationship at six levels, including *Proteobacteria*, *Gammaproteobacteria*, *Enterobacteriales*, *Enterobacteriaceae*, *Escherichia-Shigella*, and *Escherichia coli*, and their abundance was significantly higher than that of the CG (Figure 3).

### No Significant Differences in the Abundance of Intestinal Microbiota Regardless of Pathology or Gastrointestinal Involvement in Untreated DLBCL Patients

For the GCB and ABC groups, 24 fecal samples were analyzed by 16S rRNA gene sequencing. The optimized sequence was obtained through data quality control, sequential splicing, filtering, and removing the chimera. The length of the optimized sequence was mostly enriched at 380–440 bp and 2,442,128 reads were obtained and 39,864 reads were obtained per one sample. In 24 sequenced stool samples, 750 OTUs were identified. There were 559 and 636 OTUs in the GCB and ABC, respectively, with 445 identical in both groups.

For GI and NGI group, 25 fecal samples were analyzed by 16S rRNA gene sequencing. The length of the optimized sequence was mostly enriched at 380–440 bp, and 2,475,995 reads were obtained and 39,864 reads were obtained per one sample. In 25 sequenced stool samples, 763 OTUs were identified. There were 529 and 690 OTUs in the GI and NGI groups, respectively, with 456 identical in both groups.

Shannon-Wiener curve assessment for GCB and ABC and GI and NGI groups showed that the curves tend to be flat, indicating that the amount of fecal samples sequenced were sufficient to reflect the most microbial information in the samples. No significant difference between the GCB and ABC groups was found by α-diversity analysis as shown by chao1 index *P* = 0.64, Shannon index *P* = 0.40, observed_species index *P* = 0.63, and PD_whole_tree index *P* = 0.63. Similarly, there was no difference between the GI and NGI groups as shown by chao1 index *P* = 0.62, Shannon index *P* = 0.22, observed_species index *P* = 0.82, and PD_whole_tree index *P* = 0.82. In addition, no significant difference was found between the GCB and ABC (*P* = 0.32) and GI and NGI groups (*P* = 0.16) by β-diversity analysis.

PICRUSt was used to predict the microbial function based on the 16S rRNA gene sequencing data from all 51 fecal samples. Thiamine metabolism and phenylalanine, tyrosine, and tryptophan biosynthesis functions were altered. More specifically, the EG was significantly lower than the CG (*P* = 0.00006 and 0.00017, respectively).

Further analysis indicated that there was no significant correlation between age, LDH, IPI score, and the measured gut microbiota (*P* = 0.826, 0.236, and 0.88, respectively).

## Discussion

The aim of this study was to explore through 16S rRNA gene sequencing from fecal samples whether the gut microbiota was changed in untreated DLBCL patients. Significant changes in gut microbiota between EG and CG were found by β-diversity analysis. From phylum to families, the abundance of *Proteobacteria* (phylum), *Gammaproteobacteria* (class), *Enterobacteriales* (order), and *Enterobacteriaceae* (family) was significantly higher in the EG than CG. Similarly at the genus level, the abundance of *Escherichia-Shigella*, *Enterococcus*, *Veillonella*, and *Prevotella-2* was significantly higher but abundance of *Allisonella*, *Lachnospira*, and *Roseburiain* was lower in the EG than CG. At the species level, abundance of *E. coli* and *C. butyricum* was increased significantly while the abundance of *B. fragilis* and *L. garvieae* was decreased in the EG. These data collectively demonstrated that the gut microbiota in DLBCL has changed considerably.

An important discovery was that the dominant gut microbiota in untreated DLBCL patients showed a continuous evolutionary relationship at six levels (phylum, class, order, family, genus, and species). *Proteobacteria*, *Gammaproteobacteria*, *Enterobacteriales*, *Enterobacteriaceae*, *Escherichia-Shigella*, and *E. coli*. Each level of microbiota produces proteins that are involved in different cell functions. Studies have shown that *Enterobacteriales* can produce 1,548 core proteins and 53 ribosomal proteins at the order level, and five of these proteins are produced only by *Enterobacteriales*, namely, L-arabinose isomerase, elongation factor P-like protein YeiP, peptide ABC transporter permease, pyrophosphatase, and hypothetical protein (Adeolu et al., 2016). Thus, the molecular characteristics of each microbial level can be identified through the division of biological evolutionary trees, as well as the specific functions of the corresponding microbiota, and the biological markers of intestinal microbes in the untreated DLBCL can be established.

Colibactin and cytolethal distending toxin are both produced by *E. coli* and display DNAse activity. When released in the gastrointestinal epithelium, the toxins generate DNA double-strand breaks within the epithelial cells, allowing for genomic mutation to arise, and finally leading to tumor formation (Lara-Tejero and Galán, 2000). *Proteobacteria* have been shown to be associated with intestinal inflammatory diseases (Rehman et al., 2010), such as Crohn’s disease and ulcerative colitis. Although increased *Proteobacteria* may be associated with B cell differentiation (Mirpuri et al., 2014), the underlying mechanism remains elusive. We found that *Proteobacteria* was the dominant microbiota in the untreated DLBCL patients, and a continuous biological evolutionary relationship with other levels of dominant flora was observed. Therefore, *Proteobacteria* phylum is likely to play an important role in the occurrence and development of DLBCL, which lays a solid foundation to further explore whether *Proteobacteria* is a novel therapeutic target for the treatment of DLBCL.

Recent studies have confirmed that the gut microbiota cannot suppress tumors through a variety of proteins and metabolites, but it can regulate anticancer treatment through the immune system (Abid et al., 2019). Immune checkpoint inhibition (ICI) is considered a breakthrough in the field of anticancer treatment, and evidence shows that the gut microbiota of patients with cancer is a determinant of ICI response. *Proteobacteria* is related to low immunity and is associated with adverse reactions and lower survival rates in anticancer treatment and is also related to the response to ICI (Abid, 2019). Therefore, *Proteobacteria* may affect DLBCL through the immune system.

We also observed that the abundance of *B. fragilis* in DLBCL patients was significantly lower than that in the healthy controls. Previous studies have shown that *B. fragilis* can inhibit the intestinal inflammation caused by sodium dextran sulfate and the occurrence of colon tumors in mice through suppressing expression of chemokine receptor CCR5 expression (Karp et al., 2012). The relative lack of *B. fragilis* in the intestinal microbiota in DLBCL patients suggests that it inhibits the occurrence of colonic tumors and DLBCL.

The results of PICRUSt prediction of metagenome function showed that thiamine metabolism and phenylalanine, tyrosine, and tryptophan biosynthesis were decreased in DLBCL. In malignant tumor cells, pyruvate-dehydrogenase-kinase-mediated glycolysis is enhanced to maintain their rapid growth. High-dose thiamine can reduce pyruvate dehydrogenase kinase activity and therefore plays an anti-tumor role in xenograft mice (Jonus et al., 2020). In this study, the significant decreased of thiamine metabolism in DLBCL patients suggests that this disturbance of thiamine metabolism of the gut microbiota may cause abnormal glucose metabolism, leading to the occurrence and development of DLBCL although further studies on the detailed mechanism are needed. Another interesting observation is that the biosynthesis of phenylalanine, tyrosine, and tryptophan were significantly lower in the gut microbiota of DLBCL patients. Studies have shown that phenylalanine, tyrosine, and tryptophan are significantly lower in the body fluids and tissues of patients with malignant esophageal and gastric tumors (Wiggins et al., 2015), although the underlying mechanism remains unclear.

Gene mutation is one of the most important mechanisms in the pathogenesis of DLBCL. In microecological systems, material movement, energy flow, and gene transfer exist, between microorganisms and prokaryotic cells. Gene transfer, also called recombination, can be carried out by conjugation, transformation, or transduction (Treangen and Rocha, 2011). From the microbial prokaryotic cells to the mammalian eukaryotic cells, the basic structure of the DNA remains the same, making gene transfer (recombination) possible. Recent studies have shown that the DNA from eukaryotes and prokaryotes can be transferred to each other to cause various gene mutations (Dunning Hotopp et al., 2007). For example, bacterial DNA can be integrated into the human somatic genome through an RNA intermediate. Random integration of *Acinetobacter*-like DNA in the human mitochondrial genome has been demonstrated in acute myeloid leukemia samples *in vitro* (Riley et al., 2013). Thus, based on the results of the current study, we propose the likely presence of gene transfer (recombination) between gut microbiota and somatic cells in the DLBCL patients although further studies are warranted in this regard. Elongation factor P-like protein YeiP is a subtype of the elongation factor P (EF-P), which can only be produced by *Enterobacteriales* (Adeolu et al., 2016; Mohapatra et al., 2017). After lysis of *Enterobacteriales*, the DNA sequence might transfer into the human genome of B cells to cause abnormal translation of the protein (Tollerson et al., 2017).

## Conclusion and Expectation

This study confirmed that the structure of gut microflora in patients with DLBCL has changed significantly. At the levels of phylum, class, order, family, genus, and species, the dominant flora formed, in which *Proteobacteria*, *Gammaproteobacteria*, *Enterobacteria*, *Enterobacteriaceae*, *Escherichia-Shigella*, and *E. coli* showed a continuous evolutionary relationship. *Allisonella*, *Lachnospira*, *Roseburia*, *B. fragilis*, and *L. garvieae* were significantly reduced or absent at the genus and species levels. Gut microbiota plays an important role in the occurrence and development of DLBCL. These findings provide an important theoretical basis for the study of the pathogenesis of DLBCL and individualized precision treatment. We will continue to study the function, metabolites, and biomolecular characteristics of each dominant and deleted flora of DLBCL, as well as its role and mechanism in the occurrence and development of DLBCL, to identify novel biological markers for better diagnosis and treatment of DLBCL.

## Data Availability Statement

The datasets presented in this study can be found in online repositories. The names of the repository/repositories and accession number(s) can be found below: https://www.ncbi.nlm. nih.gov/bioproject/690651.

## Ethics Statement

The studies involving human participants were reviewed and approved by Ethics Committee of Peking Union Hospital. The patients/participants provided their written informed consent to participate in this study.

## Author Contributions

DZ and WZ obtained funding for the study. DZ, JL, and LY designed this study. LY, WW, and YZ participated in literature searches and study selection. LY and CW extracted data, evaluated study quality, and assessed bias risk of eligible trials and carried out all statistical analyses. LY and DZ interpreted the data. LY drafted the report. DZ and WZ revised the manuscript critically. DZ had full access to all data in the study and takes final responsibility for the decision to submit for publication. All the authors contributed to resolve divergence and approved the final submitted version.

## Conflict of Interest

The authors declare that the research was conducted in the absence of any commercial or financial relationships that could be construed as a potential conflict of interest.

## Footnotes

1 http://www.ncbi.nlm.nih.gov/bioproject/690651
