# Supplementary material for: Gut Microbiota in Untreated Diffuse Large B Cell Lymphoma Patients
Source: Front Microbiol. 2021 Apr 13;12:646361. doi: 10.3389/fmicb.2021.646361 (PMC8076791; doi:10.3389/fmicb.2021.646361)
Supplement: Supplementary file 1 [file Data_Sheet_1.ZIP › picrust.docx]

CG EG 95% confidence intervals

Thiamine metabolism Phenylalanine, tyrosine and tryptophan biosynthesis Chlorocyclohexane and chlorobenzene degradation

Pantothenate and CoA biosynthesis

Amoebiasis ABC transporters

Non-homologous end-joining Carbon fixation in photosynthetic organisms

Zeatin biosynthesis Biosynthesis of unsaturated fatty acids

Vitamin B6 metabolism Lysine degradation

One carbon pool by folate Tryptophan metabolism Aminobenzoate degradation

Protein export Bacterial secretion system Pyruvate metabolism Riboflavin metabolism

Alanine, aspartate and glutamate metabolism

Caprolactam degradation Fatty acid degradation Glycolysis / Gluconeogenesis Two-component system

NOD-like receptor signaling pathway

Dioxin degradation Nitrotoluene degradation Benzoate degradation African trypanosomiasis Cell cycle - Caulobacter

Glycerophospholipid metabolism Synthesis and degradation of ketone bodies Biosynthesis of siderophore group nonribosomal pep...

Folate biosynthesis Butanoate metabolism Propanoate metabolism RNA degradation Tyrosine metabolism

mRNA surveillance pathway Taurine and hypotaurine metabolism Ascorbate and aldarate metabolism

Methane metabolism

6.49e-5

1.73e-4

8.33e-4

1.18e-3

1.43e-3

1.49e-3

1.80e-3

1.88e-3

2.48e-3

3.35e-3

3.60e-3

4.19e-3

7.29e-3

0.012

0.012

0.012

0.012

0.013

0.013

p-value (corrected)

0.014

0.014

0.014

0.014

0.014

0.015

0.017

0.020

0.021

0.024

0.024

0.025

0.027

0.036

0.037

0.039

0.040

0.042

0.044

0.044

0.046

0.049

0.049

0.0 1.9


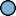

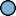

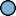

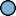

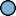

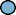

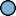

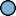

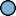

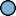

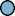

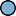

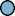


Mean proportion (%)

0.3 0.2 0.1 0.0 0.1 0.2 0.3

Difference in mean proportions (%)
