# Supplementary figures and images for: Gut Microbiota in Untreated Diffuse Large B Cell Lymphoma Patients
Source: Front Microbiol. 2021 Apr 13;12:646361. doi: 10.3389/fmicb.2021.646361 (PMC8076791; doi:10.3389/fmicb.2021.646361)

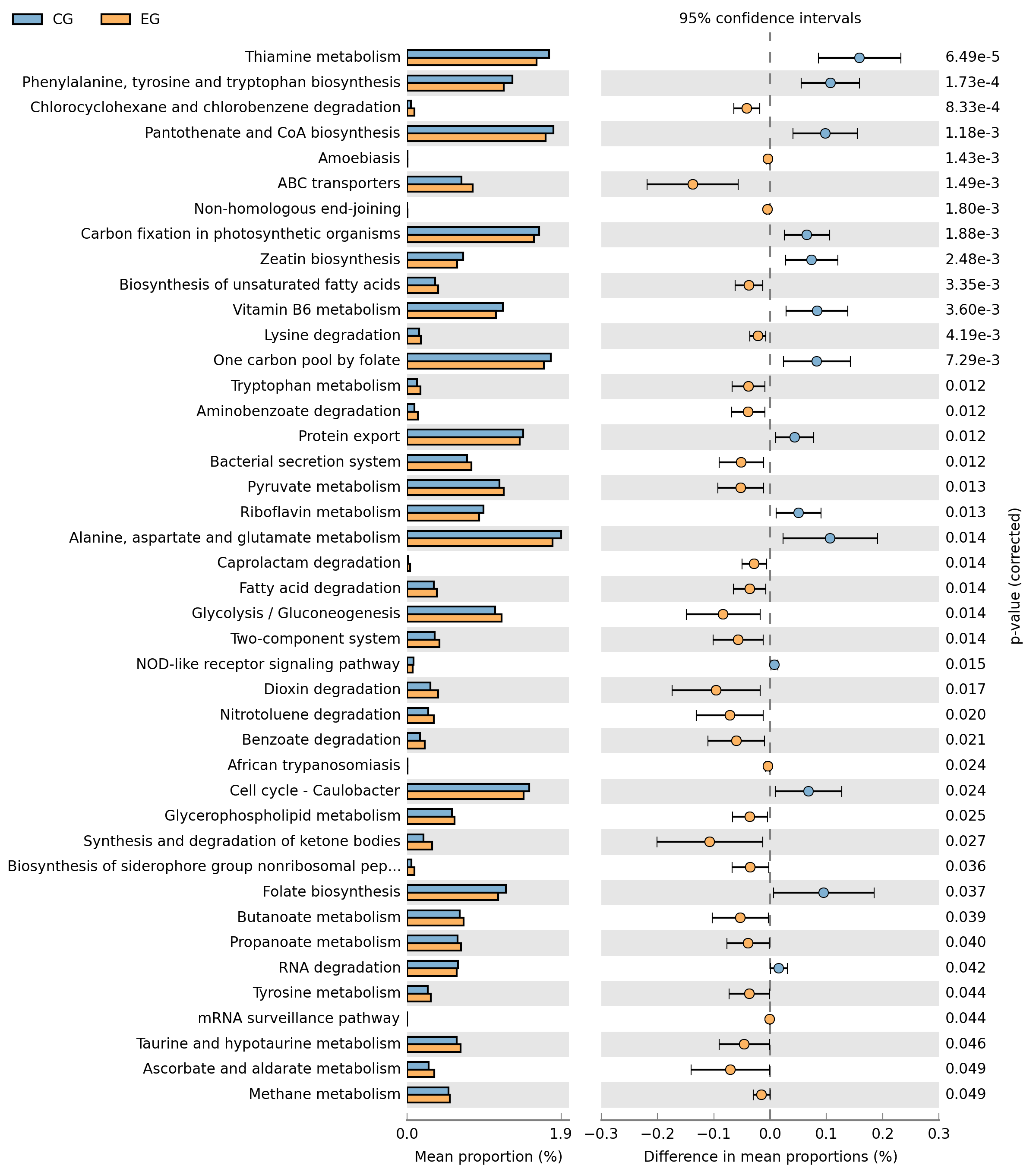

Supplement: Supplementary file 2 [file Data_Sheet_2.ZIP › supplementary tables/PICRUST(EG and CG).png]

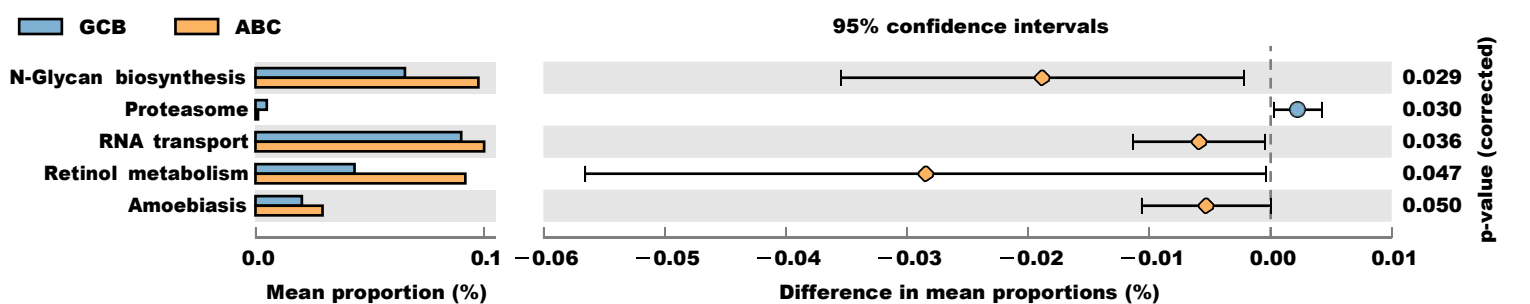

Supplement: Supplementary file 2 [file Data_Sheet_2.ZIP › supplementary tables/PICRUSTú¿GCB AND ABCú⌐.pdf]

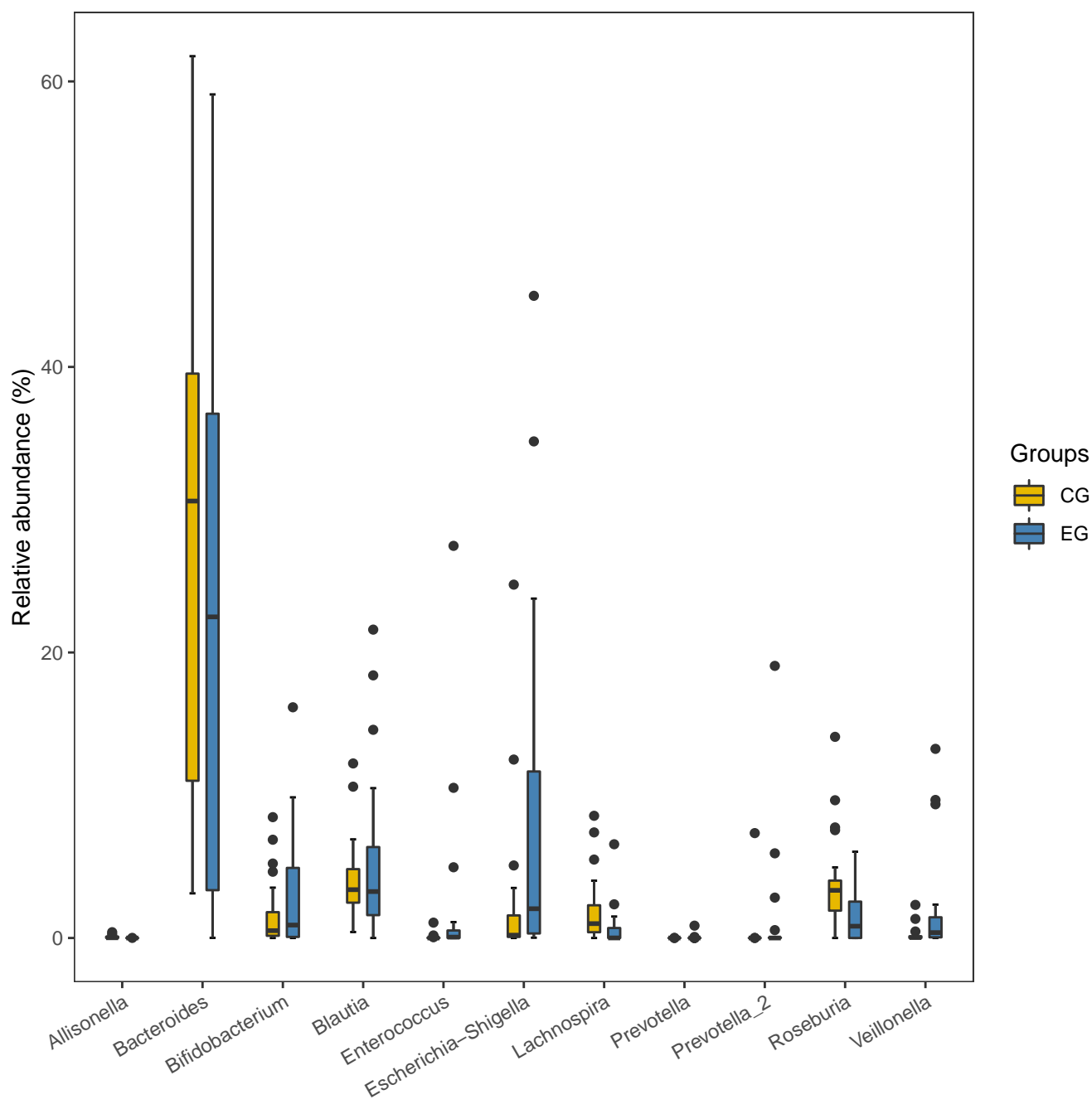

Supplement: Supplementary file 2 [file Data_Sheet_2.ZIP › supplementary tables/data of dominant bacteria (EG and CG)_G.pdf]

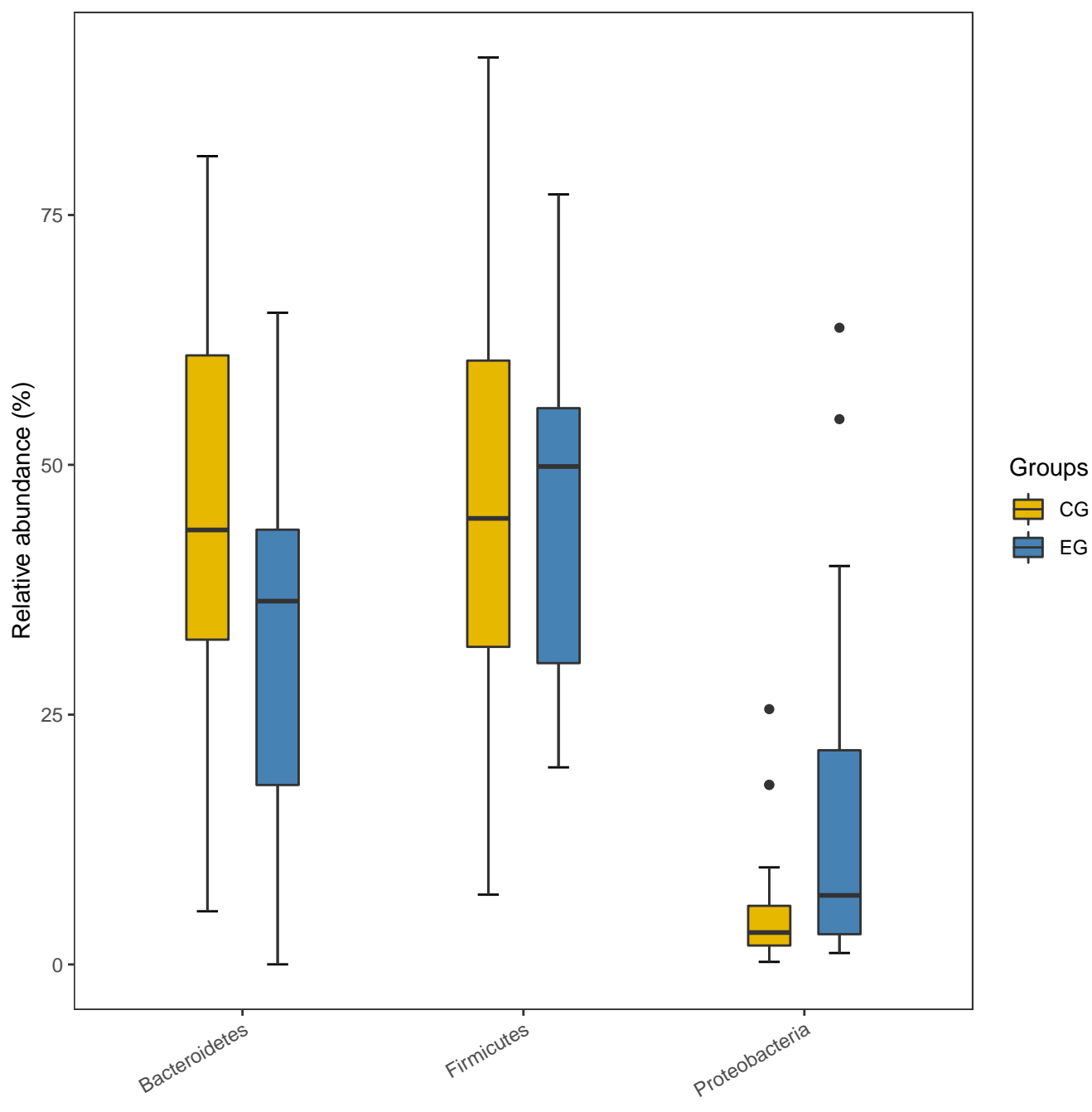

Supplement: Supplementary file 2 [file Data_Sheet_2.ZIP › supplementary tables/data of dominant bacteria (EG and CG)_P.pdf]

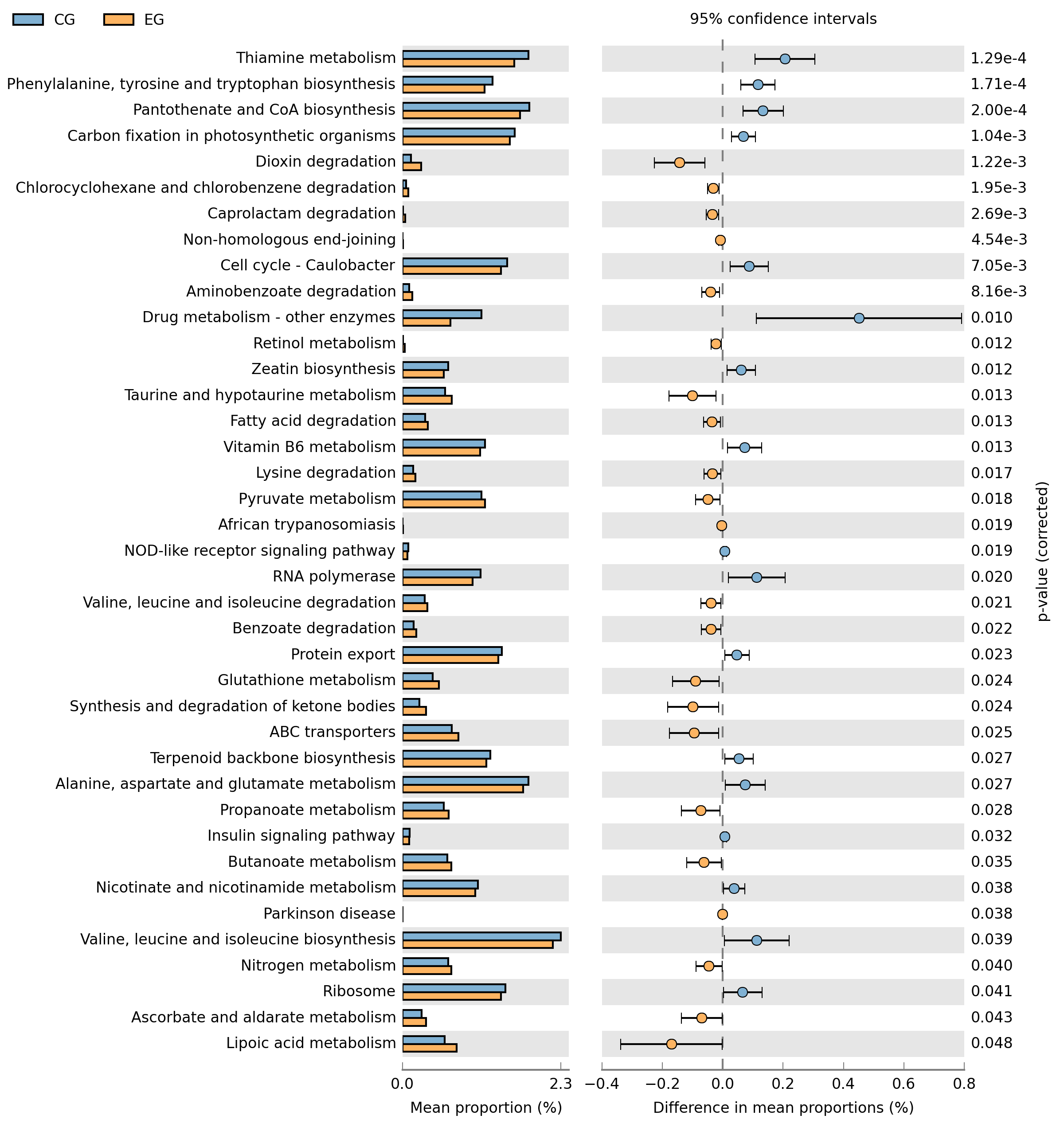

Supplement: Supplementary file 2 [file Data_Sheet_2.ZIP › supplementary tables/qimme2 and picrust2/CG-EGú¿PICRUSt2ú⌐.png]

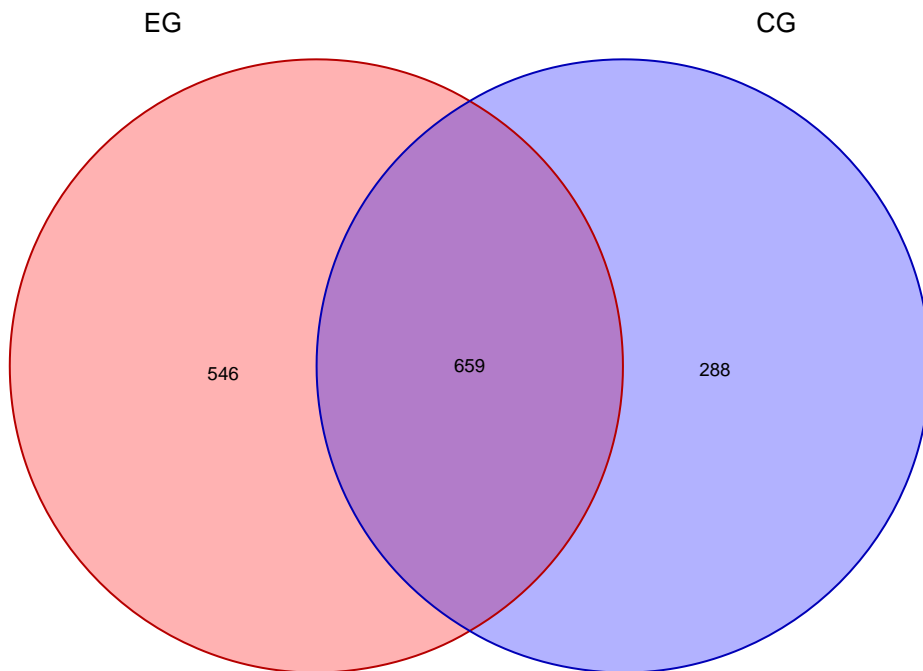

Supplement: Supplementary file 2 [file Data_Sheet_2.ZIP › supplementary tables/qimme2 and picrust2/venn1.pdf]

**chao1**

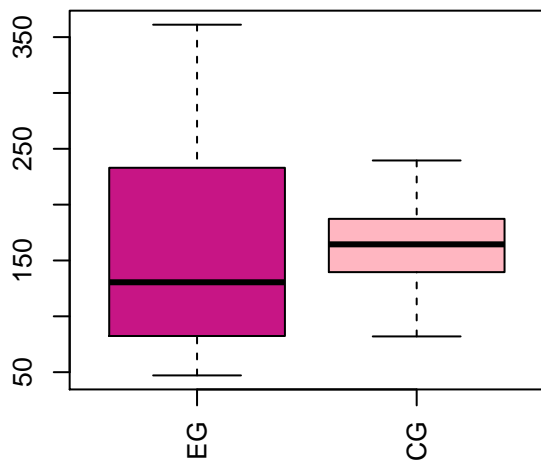

**observed\_species**

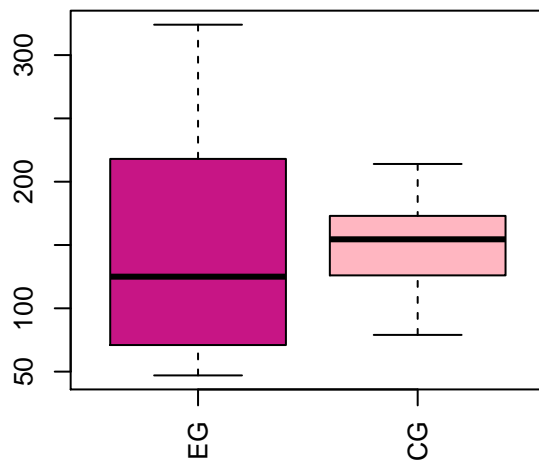

**PD\_whole\_tree**

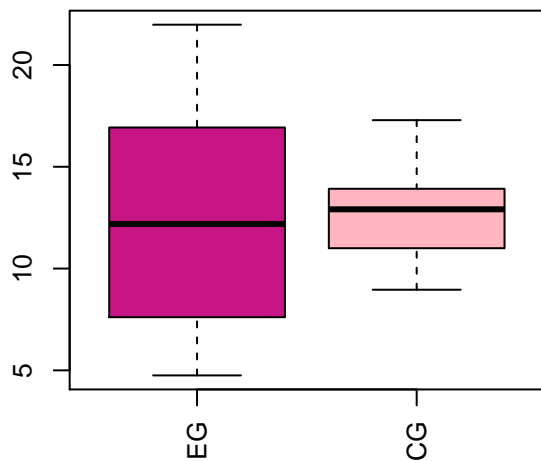

**shannon**

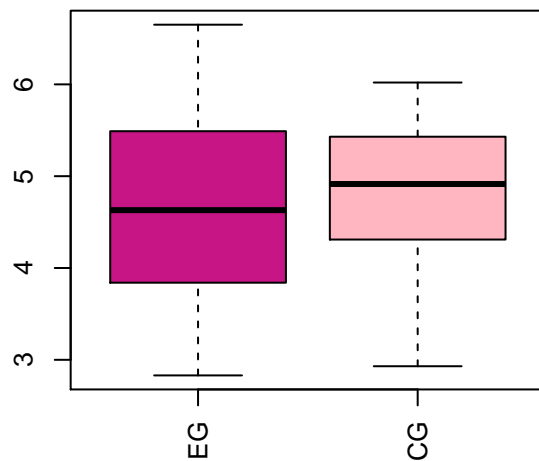

Supplement: Supplementary file 2 [file Data_Sheet_2.ZIP › supplementary tables/qimme2 and picrust2/a┴ analysis/alpha(EG+CG).pdf]

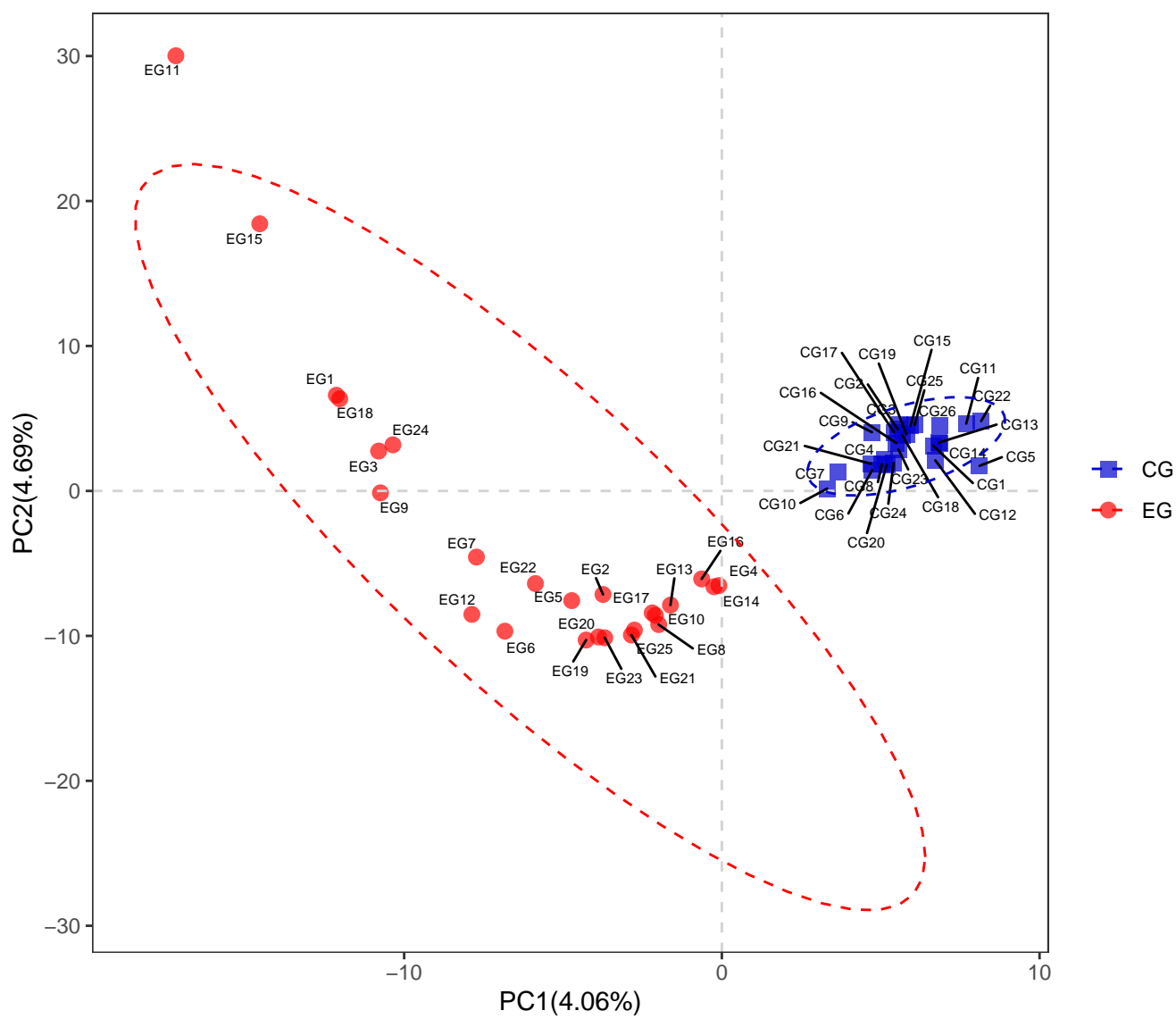

Supplement: Supplementary file 2 [file Data_Sheet_2.ZIP › supplementary tables/qimme2 and picrust2/a┬ analysis/EG+CG_PLS-DA_text.pdf]

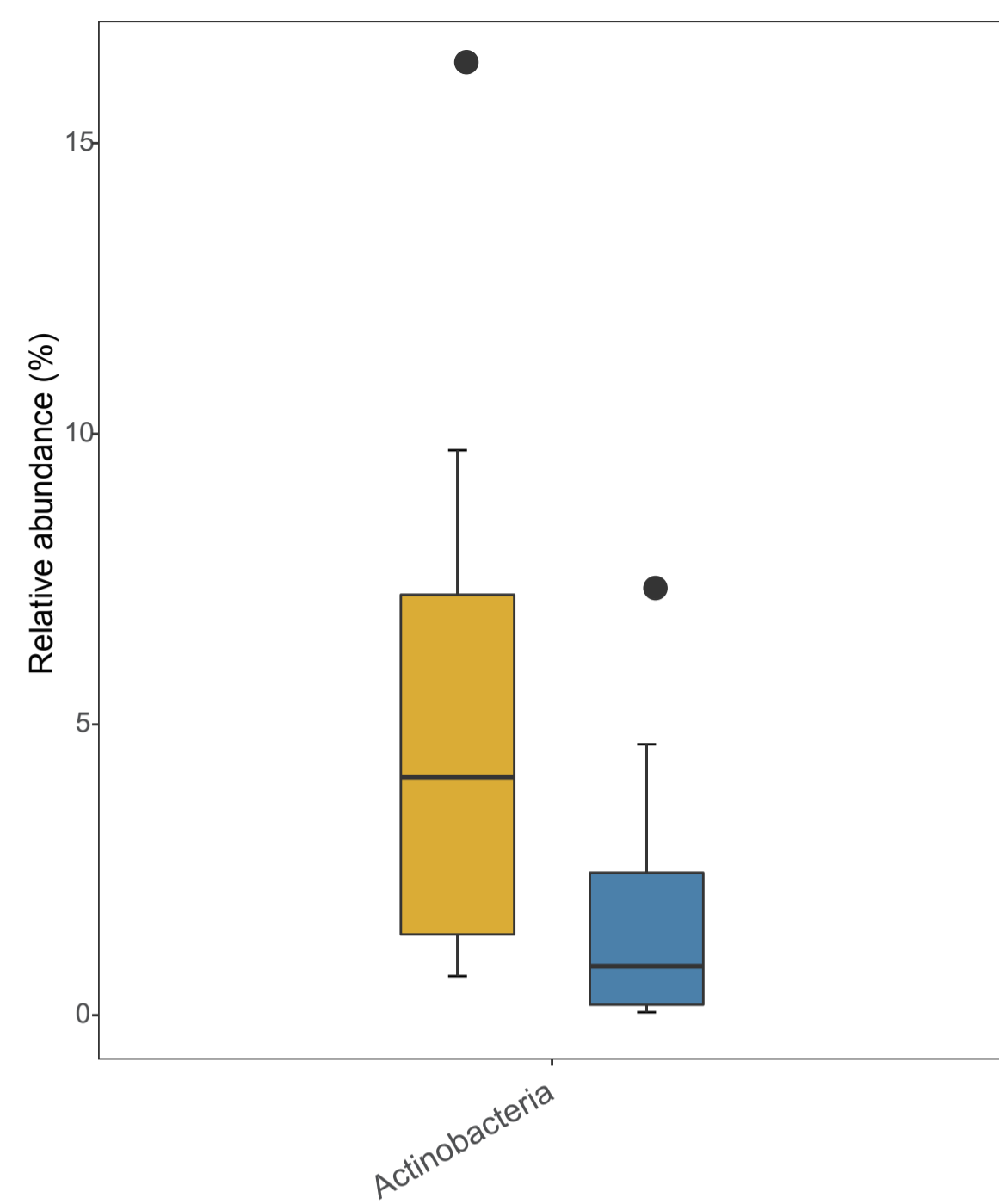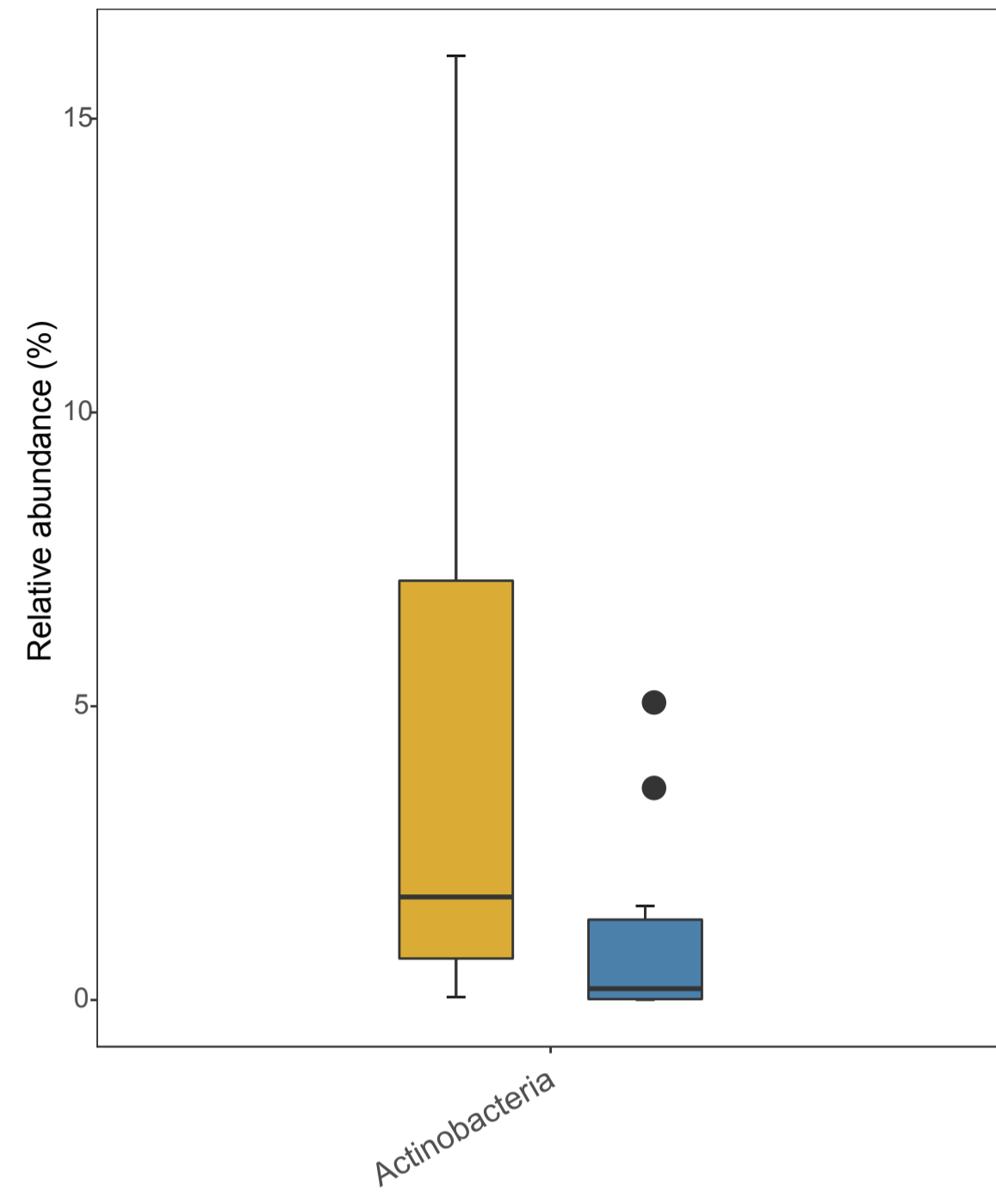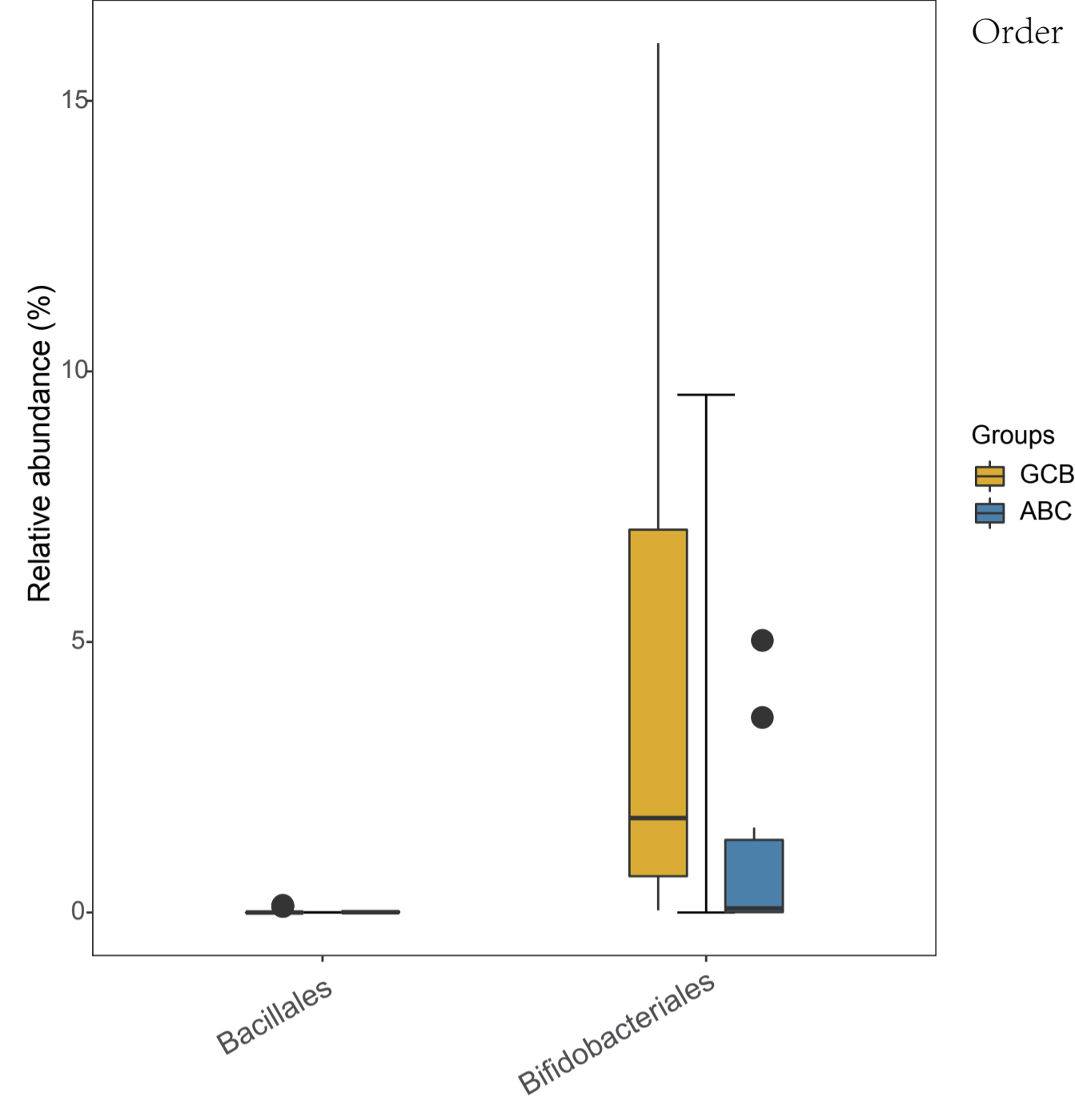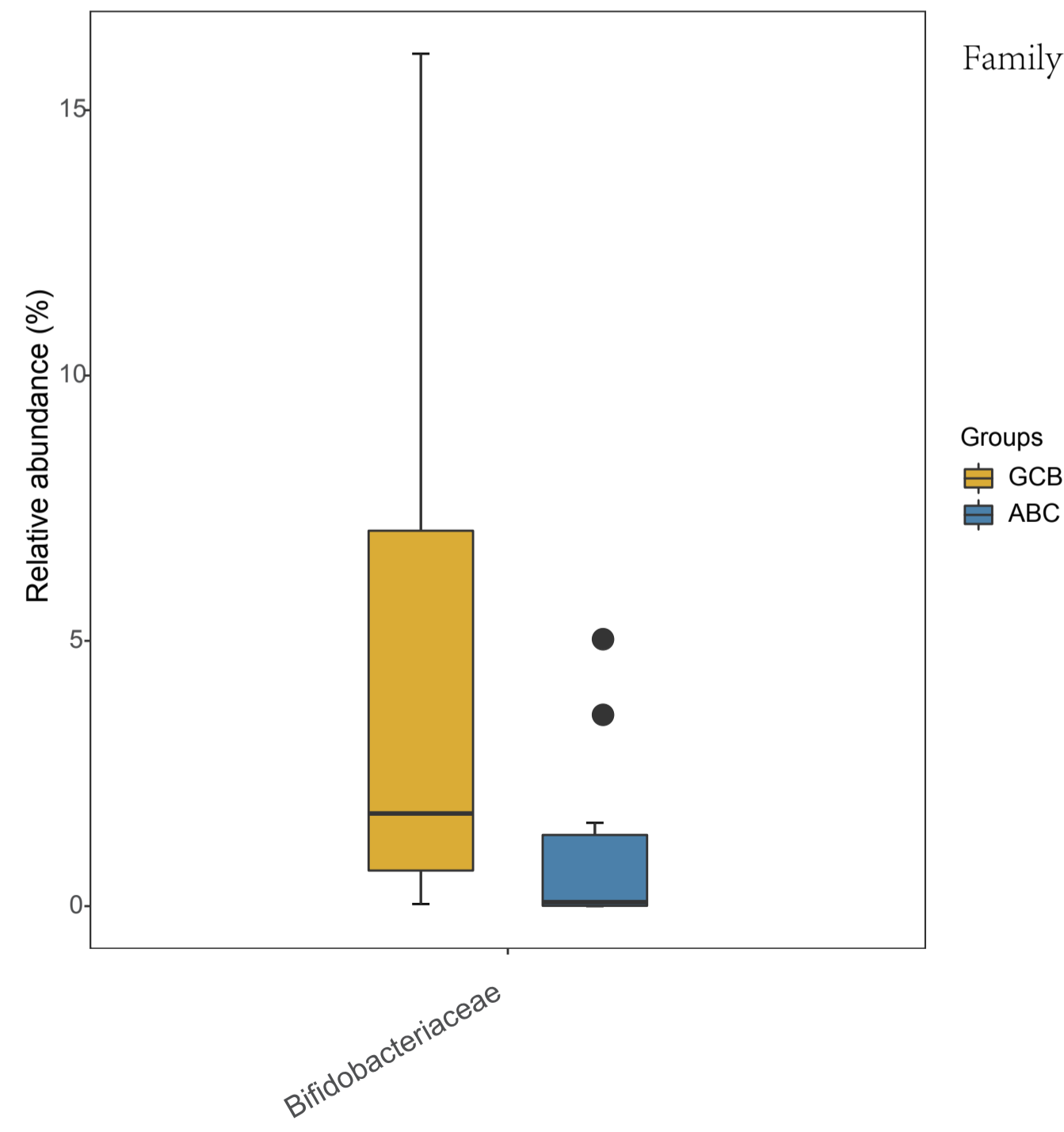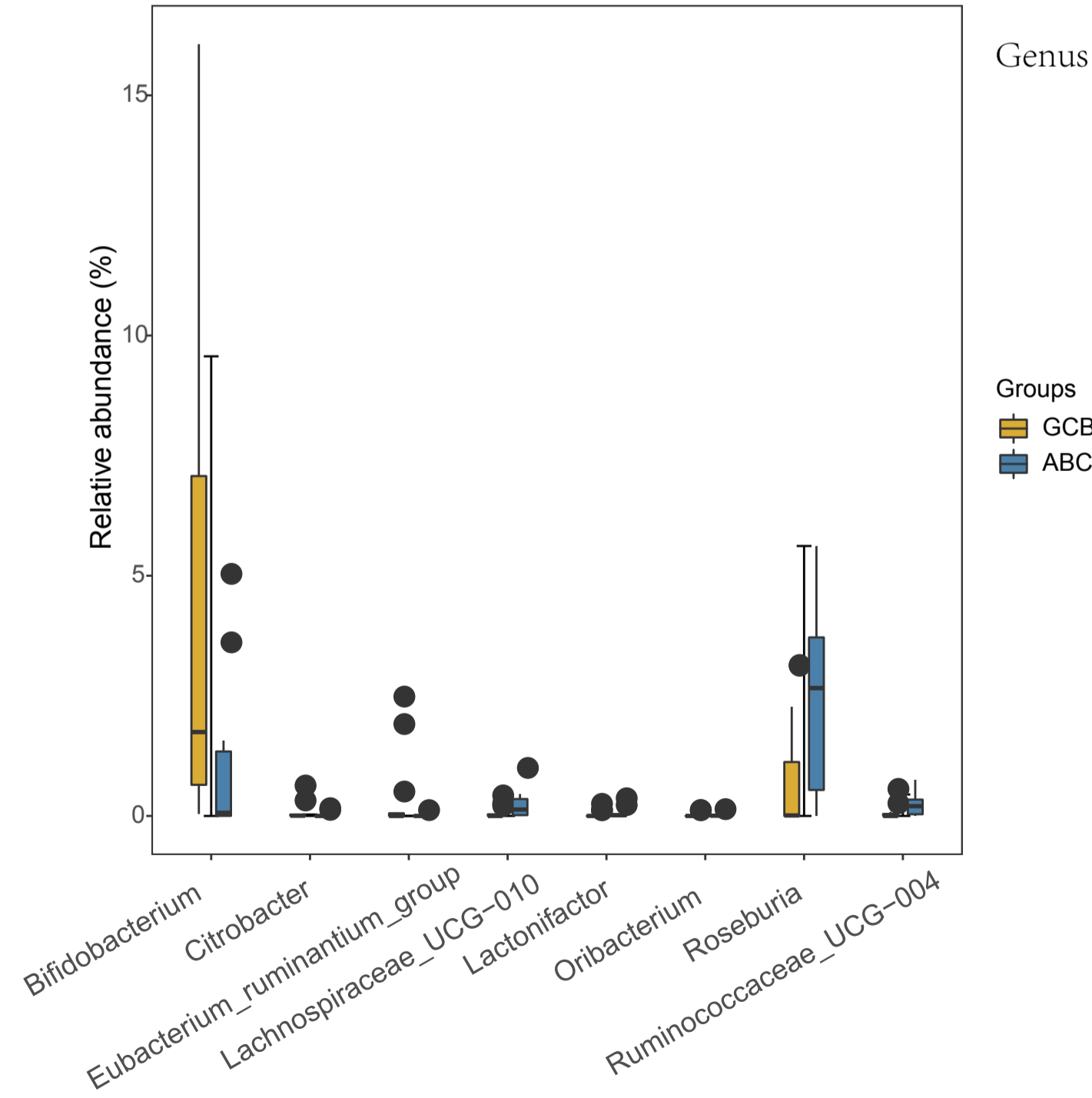

Supplement: Supplementary file 2 [file Data_Sheet_2.ZIP › supplementary tables/the result of microbiota comparison between GCB and ABC groups.pdf]

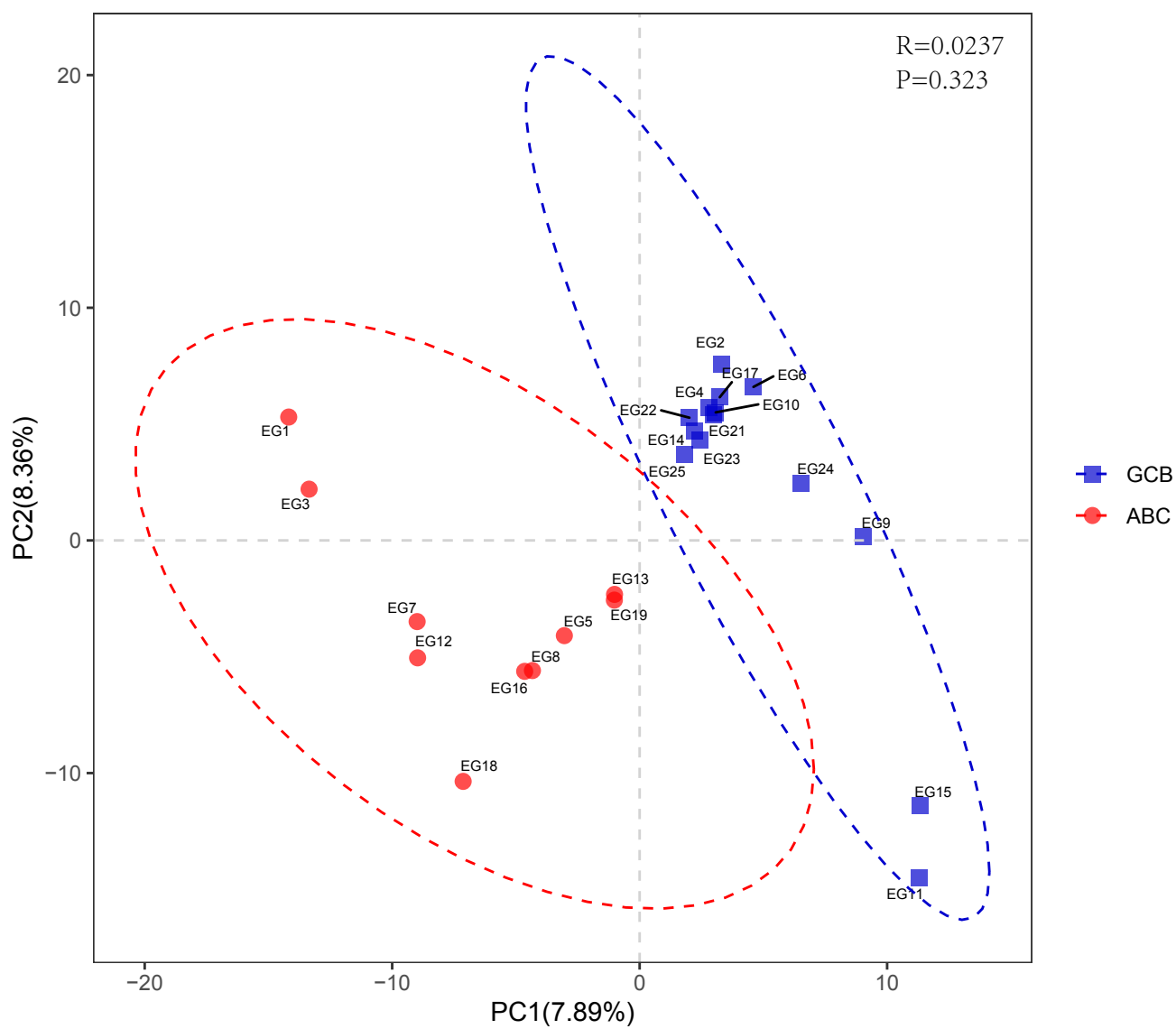

Supplement: Supplementary file 2 [file Data_Sheet_2.ZIP › supplementary tables/a┬-analisis of microbiota comparison between GCB and ABC groups_PLS-DA_text.pdf]
